# High prevalence of wild‐type transthyretin cardiac amyloidosis in older adults with carpal tunnel syndrome, heart failure or increased left ventricular mass: The CAPTURE study

**DOI:** 10.1002/ejhf.70030

**Published:** 2025-09-01

**Authors:** Alberto Aimo, Giuseppe Vergaro, Maria Concetta Pastore, Daniela Tomasoni, Vincenzo Castiglione, Riccardo Saro, Elisa Zaro, Antonio Maria Sammartino, Elisa Giacomin, Matteo Serenelli, Alberto Cipriani, Aldostefano Porcari, Andrea Di Lenarda, Marco Metra, Gianfranco Sinagra, Matteo Cameli, Marco Merlo, Michele Emdin

**Affiliations:** ^1^ Interdisciplinary Center of Health Sciences, Scuola Superiore Sant'Anna Pisa Italy; ^2^ Cardiology Division, Fondazione Toscana Gabriele Monasterio Pisa Italy; ^3^ Division of Cardiology, Department of Medical Biotechnologies University of Siena Siena Italy; ^4^ Cardiology, ASST Spedali Civili di Brescia, Department of Medical and Surgical Specialties, Radiological Sciences, and Public Health University of Brescia Brescia Italy; ^5^ European Reference Network for Rare, Low Prevalence and Complex Diseases of the Heart (ERN GUARD‐Heart); ^6^ Azienda Sanitaria Universitaria Giuliano‐Isontina (ASUGI) University of Trieste Trieste Italy; ^7^ Cardiologic Center University of Ferrara Ferrara Italy; ^8^ Department of Cardio‐Thoraco‐Vascular Sciences and Public Health University of Padua Padua Italy; ^9^ Cardiology Unit University Hospital of Padua Padua Italy

Amyloidosis is characterized by the extracellular deposition of amyloidogenic proteins that cause tissue dysfunction, organ failure, and death.^1^ Transthyretin (TTR) and light‐chains of immunoglobulins account for most forms of amyloid cardiomyopathy (ATTR‐ and AL‐CM, respectively).^2^ ATTR‐CM predominantly affects older adult males.^3^, ^4^, ^5^ Its most common form, wild‐type (wt) ATTR‐CM, is believed to be caused by ageing‐related changes that promote the dissociation of the TTR tetramer into its monomeric subunits, leading to their tissue accumulation. Autopsy studies have revealed cardiac amyloid deposits in 25% of individuals aged ≥85 years,^6^ and in as many as 43% of individuals aged ≥75 years.^7^ Although amyloid deposition does not always become clinically evident, these data suggest that ATTR‐CM remains substantially under‐diagnosed in the elderly.

Accurately defining the prevalence of ATTR‐CM in older adults is becoming ever more critical as populations age and multiple disease‐modifying therapies enter clinical practice. In the Italian multicentre AC‐TIVE study, including patients aged ≥55 years with at least one red flag on echocardiography, the prevalence of ATTR‐CM was 24% among subjects with suspected amyloid CM.^8^ The CATCH study, a prospective screening of unselected subjects aged 65–90 years, involved the search for a monoclonal protein and bone scintigraphy in subjects with at least one red flag, and found a prevalence of ATTRwt‐CM of 0.46%.^9^

The CAPTURE study (Cardiac Transthyretin Amyloidosis in the Elderly: Unmasking its Prevalence in Individuals with Cardiac Hypertrophy, Heart Failure, and Carpal Tunnel Syndrome) was conceived to investigate an elder population, as in CATCH, while placing additional emphasis on the clinical red flag of carpal tunnel syndrome (CTS)^10^ in addition to the presence of heart failure (HF) and/or left ventricular (LV) hypertrophy. To be eligible, participants had to meet all of the following criteria: age ≥65 years; history of mono‐ or bilateral CTS, whether surgically treated or not; history of HF and/or increased LV mass index relative to current echocardiographic cut‐offs,^11^ regardless of possible causes (e.g. arterial hypertension); no prior diagnosis of amyloid CM. The study involved the following referral centres for amyloid CM in Italy: Pisa, Trieste, Brescia, Ferrara, Siena, Padua. Each centre established a collaboration with general practitioners who were asked to identify suitable patients and to refer them to the nearest amyloidosis centre for the standard diagnostic workup.^12^ A total of 1215 patients were enrolled over 2 years (70% men, age 77 ± 5 years). Their main characteristics are reported in *Table* *1*. More than half of subjects (*n* = 668, 55%) had both HF and LV hypertrophy, while 377 (31%) had LV hypertrophy but not HF, and 170 (14%) had HF but not LV hypertrophy. ATTR‐CM was diagnosed in 311 patients (90% men, age 80 ± 4 years), corresponding to a 26% prevalence of ATTR‐CM. The vast majority of these patients presented with the wt form (*n* = 297, 95%), and 261 (84%) were diagnosed through the non‐invasive approach, as they presented with Perugini score 2–3 and no monoclonal protein.^12^ An additional 32 subjects (3%) were diagnosed with AL‐CM based on a tissue‐based protocol, yielding an overall prevalence of amyloid CM of 28%. The prevalence of amyloid CM was 34% in patients with HF and LV hypertrophy, 26% in those with LV hypertrophy alone, and 14% in those with HF alone.

**Table 1:** Main characteristics of the whole cohort and the subgroups with final diagnoses of amyloid transthyretin or light‐chain cardiomyopathy

Characteristics	Subjects screened (*n* = 1215)	ATTR‐CM (*n* = 311)	AL‐CM ( *n* = 32)
Age, years, mean ± SD	77 ± 5	80 ± 4	69 ± 10
Male sex, %	70	90	62
Hypertension, %	85	91	66
Diabetes, %	24	28	25
MGUS, %	30	10	80
LV hypertrophy and HF, %	55	65	45
LV hypertrophy alone, %	31	33	25
HF alone, %	14	2	30

AL‐CM, light‐chain cardiomyopathy; ATTR‐CM, amyloid transthyretin cardiomyopathy; HF, heart failure; LV, left ventricular; MGUS, monoclonal gammopathy of unknown significance.

The CAPTURE study provided novel insight on the prevalence of ATTR‐CM in older adults presenting with specific red flags, including CTS, HF and/or increased LV mass. Across Italian referral centres, 1215 participants were enrolled, and 26% were diagnosed with ATTR‐CM; most of them had the wt form. These results align with the AC‐TIVE study, which found a prevalence of ATTR‐CM of 24% among individuals aged ≥55 years whose echocardiograms suggested amyloid CM.^8^ Together, the two studies underscore the substantial disease burden in this high‐risk group and the critical need for heightened clinical surveillance and earlier diagnosis. Although much less frequent, AL‐CM was still detected in 3% of study participants. Interestingly, 11 of these patients had a history of multiple myeloma or monoclonal gammopathy of unknown significance. These haematologic disorders are often not linked to cardiac symptoms unless overt red flags are recognized, also because of the lack of specific recommendations on cardiac screening in patients with plasma cell disorders.^13^ Therefore, a diagnosis of AL‐CM can still be missed despite prior haematologic follow‐up, underscoring the value of systematic cardiac screening in high‐risk populations.

The global prevalence of amyloid CM (ATTR‐ or AL‐CM) in CAPTURE was 28%, again mirroring the 29% prevalence among suggestive echocardiograms in AC‐TIVE.^8^ LV hypertrophy seemed to drive the diagnostic yield more than HF, as demonstrated by the much higher yield of the screening in patients with LV hypertrophy (with or without HF) than in those with HF alone (41% vs. 14%). This suggests that increased LV mass should prompt screening for amyloid CM in elderly individuals with CTS even in the absence of overt HF. This is also in agreement with the current diagnostic algorithm.^12^

Several limitations must be acknowledged. Our study had an intrinsic selection bias by enrolling only subjects previously undergoing a cardiology examination resulting in a diagnosis of HF and/or LV hypertrophy. While checking that the previous cardiology exams allowed adjudicating LV hypertrophy and/or HF, we did not repeat these examinations and therefore did not reclassify patients into these categories. In many cases, the prior cardiology evaluation had apparently not even identified a possible aetiology. As a result, alternative diagnoses (e.g. hypertensive cardiomyopathy) were often unclear and not systematically recorded. Among the patients diagnosed with amyloid CM according to the current diagnostic algorithm,^12^ conditions such as long‐standing hypertension may have contributed to the HF or LV hypertrophy phenotype. The timing of CTS diagnosis was not recorded in the majority of cases, therefore this study cannot give further insight on the time from CTS diagnosis to amyloid CM development. Additionally, the number and characteristics of eligible patients who declined participation were not systematically recorded, as these individuals did not provide consent for data collection. This represents a potential source of selection bias, as their clinical profiles remain unknown. We also acknowledge the absence of an age‐matched comparator group without red flags, which would have added valuable context.

In summary, CAPTURE reinforces that amyloid CM, particularly the ATTRwt form, is common rather than exceptional in older adults who present with CTS, HF, and/or increased LV mass. The combination of a high diagnostic yield and the operational setbacks encountered in primary care emphasizes the need to streamline the process by documenting red flags in electronic health records, raising clinical suspicion (possibly through automated alerts), and establishing clear, rapid referral pathways to specialist amyloidosis centres. Implementing such measures will be crucial to shorten the diagnostic workup, initiate disease‐modifying therapy while cardiac function is still preserved, and ultimately improve outcomes in this growing patient population.
